# Correction: Gheyoh Ndzi, E.; Holmes, A. Paternal Leave Entitlement and Workplace Culture: A Key Challenge to Paternal Mental Health. *Int. J. Environ. Res. Public Health* 2023, *20*, 5454

**DOI:** 10.3390/ijerph21010101

**Published:** 2024-01-17

**Authors:** Ernestine Gheyoh Ndzi, Amy Holmes

**Affiliations:** York Business School, York St. John University, York YO31 7EX, UK; a.holmes@yorksj.ac.uk

## Addition of an Author

**Amy Holmes** was not included as an author in the original publication [1]. The corrected Author Contributions Statement appears here. The authors apologize for any inconvenience caused and state that the scientific conclusions are unaffected. The original publication has also been updated.

Conceptualization, E.G.N.; methodology, E.G.N. and A.H.; software, A.H.; validation, E.G.N. and A.H.; formal analysis, A.H.; investigation, E.G.N.; resources, E.G.N.; data curation, A.H.; writing—original draft preparation, E.G.N. and A.H.; writing—review and editing, E.G.N.; visualization, E.G.N.; supervision, E.G.N.; project administration, E.G.N. All authors have read and agreed to the published version of the manuscript.
